# Skin Photoaging and the Role of Antioxidants in Its Prevention

**DOI:** 10.1155/2013/930164

**Published:** 2013-09-12

**Authors:** Ruža Pandel, Borut Poljšak, Aleksandar Godic, Raja Dahmane

**Affiliations:** ^1^Faculty of Health Studies, University of Ljubljana, Zdravstvena pot 5, 1000 Ljubljana, Slovenia; ^2^Faculty of Medicine, University of Ljubljana, Vrazov trg 2, 1000 Ljubljana, Slovenia; ^3^Department of Dermatology, Cambridge University Hospitals, Addenbrooke's Hospital, Hills Road, Cambridge CB2 0QQ, UK

## Abstract

Photoaging of the skin depends primarily on the degree of ultraviolet radiation (UVR) and on an amount of melanin in the skin (skin phototype). In addition to direct or indirect DNA damage, UVR activates cell surface receptors of keratinocytes and fibroblasts in the skin, which leads to a breakdown of collagen in the extracellular matrix and a shutdown of new collagen synthesis. It is hypothesized that dermal collagen breakdown is followed by imperfect repair that yields a deficit in the structural integrity of the skin, formation of a solar scar, and ultimately clinically visible skin atrophy and wrinkles. Many studies confirmed that acute exposure of human skin to UVR leads to oxidation of cellular biomolecules that could be prevented by prior antioxidant treatment and to depletion of endogenous antioxidants. Skin has a network of all major endogenous enzymatic and nonenzymatic protective antioxidants, but their role in protecting cells against oxidative damage generated by UV radiation has not been elucidated. It seems that skin's antioxidative defence is also influenced by vitamins and nutritive factors and that combination of different antioxidants simultaneously provides synergistic effect.

## 1. Introduction

Unlike chronological aging, which is predetermined by individual's physiological predisposition, photoaging depends primarily on the degree of sun exposure and on an amount of melanin in the skin. Individuals who have a history of intensive sun exposure, live in sunny geographical areas, and have fair skin will experience the greatest amount of ultraviolet radiation (UVR) skin load and consequently severe photoaging [1, 2]. Clinical signs of photoaging include wrinkles, mottled pigmentation (hypo- or hyperpigmentation), rough skin, loss of the skin tone, dryness, sallowness, deep furrows, severe atrophy, telangiectasias, laxity, leathery appearance, solar elastosis, actinic purpura, precancerous lesions, skin cancer, and melanoma [3, 4]. Sun-exposed areas of the skin, such as the face, neck, upper chest, hands, and forearms, are the sites where these changes occur most often [5]. Chronological skin aging, on the other hand, is characterized by laxity and fine wrinkles, as well as development of benign growths such as seborrheic keratoses and angiomas, but it is not associated with increased/decreased pigmentation or with deep wrinkles that are characteristic for photoaging [6]. Seborrheic keratoses are regarded as best biomarker of intrinsic skin aging since thier appearance is independent on sun exposure. While intrinsically aged skin does not show vascular damage, photodamaged skin does. Studies in humans and in the albino and hairless mice showed that acute and chronic UVB irradiation greatly increases skin vascularization and angiogenesis [7, 8]. The sun is the main source of UVR and the main contributor to the photoaging. UVC radiation (100 to 290 nm) is almost completely absorbed by the ozone layer and does not affect the skin. UVB (290 to 320 nm) affects the superficial layer of the skin (epidermis) and causes sunburns. It is the most intense between 10 am and 2 pm, during summer months, does not penetrate through the glass, and accounts for 70% of a person's yearly average cumulative UVB dose. UVA (320 to 400 nm) was believed to have a minor effect on the skin, but studies showed that they penetrate deeper in the skin (e.g., about 20% at 365 nm), are more abundant in sunlight (95% of UVA and 5% of UVB), and therefore exhibit more severe damage [9, 10]. Significantly more photons in the UVA are needed to cause the same degree of damage compared to UVB since they are less energetic, but they are present in much higher quantities in sunlight and are more penetrant than in UVB [9]. Until recently, it has become evident that also infrared radiation (IR) could induce skin damage and contribute to the skin photoaging. While proton energy of IR is low, total amount of IR which reaches humans' skin accounts approximately for 54% (compared to 5–7% of UV rays). Most of the IR lies within the IR-A band (*λ* = 760 to 1440 nm), which represents approximately 30% of total solar energy, and penetrates human skin deeply compared to IR-B and IR-C, which only penetrate the upper skin layers. In comparison, IR-A penetrates the skin deeper than UV, and approximately 50% of it reaches the dermis. Molecular mechanisms of damaging effect of IR-A on the skin are attributed to induction of matrix mtalloproteinase-1, as well as to generation of reactive oxygen species (ROS). The exposure of human to environmental and artificial UVR has increased significantly in the last 50 years. This is due to an increased solar UVR as a consequence of the stratospheric ozone depletion, use of sunscreens, false believe of being well protected while exposed to sun for longer time, outdoor leisure activities, and prolonged life expectancy in industrialized countries [11].

## 2. Effects of UVR on Cells and Tissues

Studies in hairless mice demonstrated the carcinogenicity of UVR, with UVB being the most effective, followed by UVC and UVA [12]. UVB radiation is three to four orders of magnitude more effective than UVA. In none of the experiments it was possible to exclude completely a contribution of UVC, but the size of the effects observed indicate that they cannot be due to UVB alone [13]. People with a poor ability to tan, who burn easily, and have light eye and hair colour are at a higher risk of developing melanoma, basal-cell, and squamous-cell carcinomas. UVB most commonly causes cyclobutane pyrimidine dimmers. UVA, on the other hand, primarily causes DNA damage indirectly by the production of short-lived reactive oxygen species (ROS) such as singlet oxygen, superoxide, and H_2_O_2_ via endogenous photosensitizers. UVA radiation generates more phosphodiester bond breaks in DNA than would be expected by the total amount of energy directly absorbed by the DNA; therefore, it most likely causes indirect damage to DNA, which is caused by endogenous photosensitizers such as riboflavin, nicotinamide coenzymes, and rarely RNA bases [9]. Damage of the skin cells' DNA is repaired by two different mechanisms: nucleotide excision repair (NER) and base excision repair (BER). The ROS-induced DNA damage is primarily repaired by the BER system and damage caused by direct influence of UVR on DNA by the NER system. DNA damage that can be induced by UVA radiation includes pyrimidine dimmers, single-strand breaks (both are critical in UVA radiation-induced cellular lethality), and perhaps more importantly DNA protein cross-links [14–17]. On the other hand, ROS can oxidize guanine in DNA to form 8-hydroxy-7,8-dihydroguanine (8-OHdG). The frequency of this characteristic mutation in human skin increases with cumulative sun exposure and could be used as an internal marker of cumulative sun exposure [18]. OH^•^ can be added to guanine at positions 4, 5, and 8 (causing 8-OHdG) or undergoes opening of the imidazole ring, followed by one-electron reduction and protonation, to give 2,6-diamino-4-hydroxy-5-formamidopyrimidine (FAPyG) [19]. Photoexcitation of cytosine and guanine may lead to the formation of relatively rare 6-hydroxy-5,6-dihydrocytosine and 8-oxo-7,8-dihydroguanine.

A second mechanism, which requires participation of endogenous photosensitizers and oxygen, causes most of the DNA damage generated by the UVA and visible light. Singlet oxygen is likely to be mostly involved in the formation of 8-oxo-7,8-dihydroguanine that was observed within both isolated and cellular DNA. It may be expected that oxidized purine together with DNA strand breaks and pyrimidine base oxidation products is also generated with a lower efficiency through Fenton type reactions [20]. The number of different DNA modifications that are capable of producing OH^•^ appears to be over 100 [21].

Solar UVR induces a variety of photoproducts in DNA, including cyclobutane-type pyrimidine dimers, pyrimidine-pyrimidone (6-4) photoproducts, thymine glycols, cytosine damage, purine damage, DNA strand breaks, and DNA-protein cross links [22]. Substantial information on biological consequences is available only for the first two classes. Both are potentially cytotoxic and can lead to mutations in cultured cells, and there is evidence that cyclobutane-type pyrimidine dimers may be precarcinogenic lesions [13].

**Figure 1:**
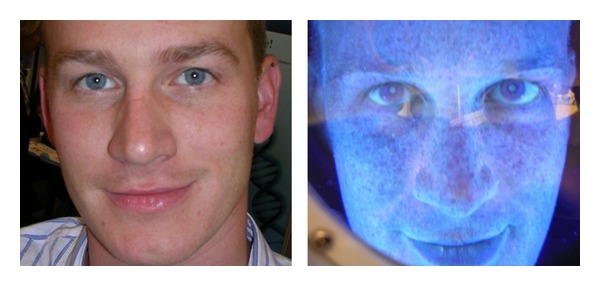
DermaView skin analyser accentuates areas of sun-damaged skin of the face.

UVR also directly or indirectly initiates and activates a complex cascade of biochemical reactions in the human skin. Besides, the UV light-induced ROS interfere with signalling pathways. On a molecular level, UVR activates cell surface receptors of keratinocytes and fibroblasts in the skin, which initiates signal transduction cascades. This, in turn, leads to a variety of molecular changes, which causes a breakdown of collagen in the extracellular matrix and a shutdown of new collagen synthesis [23]. UV-induced liberation of ROS in human skin is responsible for stimulation of numerous signal transduction pathways via activation of cell surface cytokine and growth factor receptors. UVA or UVB induce activation (sometimes via peroxides or singlet O_2_ as signalling molecules) of a wide range of transcription factors in skin cells, including factor activator protein-1 (AP-1) [10]. This can increase production of matrix metalloproteinases that can degrade collagen and other connective tissue components. For example, the UV light-induced ROS induce the transcription of AP-1. AP-1 induces upregulation of matrix metalloproteinases (MMPs) like collagenase-1 (MMP-1), stromelysin-1 (MMP-3), and gelatinase A (MMP-2), which specifically degrade connective tissue such as collagen and elastin and indirectly inhibit the collagen synthesis in the skin [24]. As indicated by their name, these zinc-dependent endopeptidases show proteolytic activity in their ability to degrade matrix proteins such as collagen and elastin [25]. Destruction of collagen is a hallmark of photoaging. The major enzyme responsible for collagen 1 digestion is matrix metalloproteinase-1 (MMP-1) [26]. Skin fibroblasts produce MMP-1 in response to UVB irradiation, and keratinocytes play a major role through an indirect paracrine mechanism involving the release of epidermal cytokine after UVB irradiation [27]. MMPs are produced in response to UVB irradiation *in vivo* and are considered to be involved in the changes in connective tissue that occur in photoaging [28]. They are associated with a variety of normal and pathological conditions that involve degradation and remodelling of the matrix [29–32]. UV rays and aging lead to excess proteolytic activity that disturbs the skin's three-dimensional integrity [33]. These proteinases are important for breaking down the extracellular matrix during chronic wound repair, in which there is reepithelialization by keratinocyte migration. Thus, MMPs are continuously involved in the remodelling of the skin after chronic damage. Photodamage also results in the accumulation of abnormal elastin in the superficial dermis, and several MMPs have been implicated in this process [33]. ROS activate cytoplasmic signal transduction pathways in resident fibroblasts that are related to growth, differentiation, senescence, and connective tissue degradation [34]. ROS activate cytoplasmic signal transduction pathways that are related to growth differentiation, senescence, transformation and tissue degradation and cause permanent genetic changes in protooncogenes and tumour suppressor genes [35]. The study of Kang et al. [36] revealed that UVA/UVB irradiation of the skin causes generation of H_2_O_2_ within 15 minutes. AP-1, which leads to increased collagen breakdown, becomes elevated and remains elevated within 24 hours following UV irradiation [37]. Decreased procollagen synthesis within eight hours of UV irradiation was demonstrated [38]. Consequently, increased collagen breakdown was demonstrated [39]. It is hypothesized that dermal breakdown is followed by repair that, like all wound repair, is imperfect. Imperfect repair yields a deficit in the structural integrity of the dermis, a solar scar. Dermal degradation followed by imperfect repair is repeated with each intermittent exposure to ultraviolet irradiation, leading to accumulation of solar scarring and ultimately visible photoaging [40]. While it may seem that the signs of photoaging appear overnight, they actually lie invisible beneath the surface of the skin for years (Figure 1). UV exposure of the skin causes oxidative stress, leading to inflammatory reactions, such as acute erythema and chronic damage. Most problematic consequences of chronic damage include premature skin aging and skin cancer [41].

## 3. Skin Antioxidants Protect against UVR

UVR exposure affects the skin antioxidants. Ascorbate, glutathione (GSH), superoxide dismutase (SOD), catalase, and ubiquinol are depleted in all layers of the UVB-exposed skin. Studies of cultured skin cells and murine skin *in vivo* have indicated that UVR-induced damage involves the generation of ROS and depletion of endogenous antioxidants [42]. In the study by Shindo et al. [43], enzymatic and nonenzymatic antioxidants in the epidermis and dermis and their responses to ultraviolet light of hairless mice were compared. Mice were exposed to solar light and subsequently examined for UV-induced damage of their skin. After irradiation, epidermal and dermal catalase and SOD activities were greatly decreased. Alpha-tocopherol, ubiquinol 9, ubiquinone 9, ascorbic acid, dehydroascorbic acid, and reduced GSH decreased in both epidermis and dermis by 26% to 93%. Oxidized GSH showed a slight nonsignificant increase. Because the reduction in total ascorbate and catalase was much more prominent in epidermis than dermis, the authors concluded that UV light is more damaging to the antioxidant defences in the epidermis than in the dermis. Many other studies confirmed that acute exposure of human skin to UVR *in vivo* leads to oxidation of cellular biomolecules that could be prevented by prior antioxidant treatment. There have been many studies performed where different antioxidants or combinations of antioxidants and different phytochemicals were tested in order to find evidence against ROS-induced damage. Some of them are presented in Tables 1 and 2.

## 4. Endogenous Skin Antioxidants

Skin has a network of protective antioxidants. They include endogenous enzymatic antioxidants such as GSH peroxidase (GPx), SOD, and catalase and nonenzymatic low-molecular-weight antioxidants such as vitamin E isoforms, vitamin C, GSH, uric acid, and ubiquinol [43]. All the major antioxidant enzymes are present in the skin, but their roles in protecting cells against oxidative damage generated by UV radiation have not been elucidated. In response to the attack of ROS, the skin has developed a complex antioxidant defence system including, among others, the manganese-superoxide dismutase (MnSOD). MnSOD is the mitochondrial enzyme that disposes of superoxide generated by respiratory chain activity. Of all electrons passing down the mitochondrial respiratory chain, it is estimated that 1% to 2% are diverted to form superoxide (although recent studies claim that this amount is even less); thus, production of hydrogen peroxide occurs at a constant rate due to MnSOD activity. MnSOD dismutates the superoxide anion (O_2_^•^^−^) derived from the reduction of molecular oxygen to hydrogen peroxide (H_2_O_2_), which is detoxified by GSH peroxidase to water and molecular oxygen. The study of Poswig et al. [44] revealed that adaptive antioxidant response of MnSOD following repetitive UVA irradiation can be induced. The authors provide evidence for the increasing induction of MnSOD upon repetitive UVA irradiation that may contribute to the effective adaptive UVA response of the skin during light hardening in phototherapy. The study of Fuchs and Kern showed that acute UV exposures lead also to changes in GSH reductase and catalase activity in mouse skin but insignificant changes in SOD and GSH peroxidase [45]. The study of Sander et al. [46] confirmed that chronic and acute photodamage is mediated by depleted antioxidant enzyme expression and increased oxidative protein modifications. Biopsies from patients with histologically confirmed solar elastosis, from non-ultraviolet-exposed sites of age-matched controls, and from young subjects were analysed. The antioxidant enzymes catalase, copper-zinc superoxide dismutase, MnSOD, and protein carbonyls were investigated. Whereas overall expression of antioxidant enzymes was very high in the epidermis, low baseline levels were found in the dermis. In photoaged skin, a significant depletion of antioxidant enzyme expression was observed within the stratum corneum and in the epidermis. Importantly, an accumulation of oxidatively modified proteins was found specifically within the upper dermis of photoaged skin. Upon acute ultraviolet exposure of healthy subjects, depleted catalase expression and increased protein oxidation were detected. Exposures of keratinocytes and fibroblasts to UVB, UVA, and H_2_O_2_ led to dose-dependent protein oxidation confirming *in vivo* results. 

Not all skin cells are exposed to the same level of oxidative stress. It was found that keratinocytes utilize as much oxygen as fibroblasts, even though maximal activities of the respiratory chain complexes are two- to five-fold lower, whereas expression of respiratory chain proteins is similar. Superoxide anion levels are much higher in keratinocytes, and keratinocytes display much higher lipid peroxidation level and a lower reduced glutathione/oxidized glutathione ratio [47]. 

It can be concluded that oxidative stress is a problem of skin cells and that endogenous as well as exogenous antioxidants could play an important role in decreasing it.

## 5. Compounds Derived from the Diet with Photoaging/Damage Protective Effects 

Natural antioxidants are generally considered to be beneficial fruit and vegetable components. It seems that skin's antioxidative defence is also influenced by nutritive factors. Besides vitamins A, C, and E, *η*-3 fatty acids certain nonvitamin plant-derived ingredients might have beneficial effect on skin aging, skin sun protection, or skin cancer. The laboratory studies conducted in animal models suggest that many plant compounds have the ability to protect the skin from the adverse effects of UVR. The proliferation of products, however, can cause confusion among consumers, who often ask their dermatologists for advice as to which antiaging products they should choose. Ideally, the antiaging claims of cosmeceutical formulations and their components should be demonstrated in controlled clinical trials [48], but there is a lack of such studies due to their high costs. Since cosmeceutical products are claiming that they therapeutically affect the structure and function of the skin, it is rational and necessary to hold them to specified scientific standards that substantiate efficacy claims [49].

Many studies have found that vitamin C can increase collagen production, protect against damage from UVA and UVB rays, correct pigmentation problems, and improve inflammatory skin conditions [50] (Table 1). 

Topical retinoids remain the mainstay for treating photoaging given their proven efficacy in both clinical and histological outcomes. The application of retinoids might not only clinically and biochemically repair photoaged skin, but their use might also prevent photoaging [102]. Retinoid-mediated improvement of photoaging is associated with increased collagen I synthesis [103], reorganization of packed collagen fibres [104], and increased number of type VII anchoring fibrils [105]. However, up to 92% of subjects who used tretinoin in various clinical studies have reported “retinoid dermatitis,” that is, erythema and scaling at the site of application [106, 107]. Irritation can be minimized by reducing dose and frequency of treatments.

It seems that the biochemistry of CoQ10 may inhibit the production of IL-6, which stimulates fibroblasts in dermis by paracrine manner to upregulate MMPs production, and contribute to protecting dermal fibrous components from degradation, leading to rejuvenation of wrinkled skin [108]. It was reported that CoQ10 strongly inhibits oxidative stress in the skin induced by UVB via increasing SOD2 and GPx [109]. It was reported that it is considered that CoQ10 appears to have also a cutaneous healing effect *in vivo* [110]. 

Green tea polyphenols have received attention as protective agents against UV-induced skin damage. Analysis of published studies demonstrates that green tea polyphenols have anti-inflammatory and anticarcinogenic as well as anti-aging properties. These effects appear to correlate with antioxidant properties of green tea polyphenols, which could be used as new photoprotection agents (Table 1).

A number of experimental studies indicate protective effects of beta-carotene against acute and chronic manifestations of skin photodamage. However, most clinical studies have failed to convincingly demonstrate its beneficial effects so far. Studies on skin cells in culture have revealed that beta-carotene acts not only as an antioxidant but also has unexpected prooxidant properties [111]. For this reason, further studies with focus on *in vivo β*-carotene-induced prooxidative properties and its relevance on human health are needed. Another problem represents the dosage. Although studies convincingly showed that vitamin supplementation effectively protects the skin against sunburn, the doses of vitamins used were generally much higher than amounts generally ingested from habitual diets [112]. Additionally, it was shown that the combination of different antioxidants applied simultaneously can provide a synergistic effect [50]. Antioxidants are most effective when used in combination (Table 2). Vitamin C regenerates vitamin E, and selenium and niacin are required to keep glutathione in its active form. It has been demonstrated that vitamin C can regenerate *α*-tocopherol from its chromanoxyl radical [113] and that the vitamin C radical may be recycled by GSH nonenzymatically under slightly acidic conditions [114] that are present in the stratum corneum [115]. Werninghaus et al. [116] reported that vitamin E given orally at 400 IU/day for a period of six months affords no significant increase in UV protection. Similarly, in a study with 12 volunteers, vitamin C given at 500 mg/day over eight weeks had no effect on the UV-induced erythemal response [85], indicating again the importance of antioxidants to be supplemented together to obtain the synergistic effect.

## 6. Conclusion

Studies (usually performed on skin cells in vitro or on animal models) suggest that oral uptake of selected micronutrients and phytochemicals can provide photoprotection of human skin [117]. Nevertheless, photoprotection can only be achieved if an optimal pharmacological dose range is reached in the human skin due to well-known prooxidative reactions of antioxidants, for example, in the case of excessive carotenoid concentrations (Table 3). Nevertheless, research is continuously demonstrating that various phytopharmaceuticals offer significant protection against different diseases and skin aging. The primary treatment of photoaging is photoprotection, but secondary treatment could be achieved with the use of antioxidants and some novel compounds such as polyphenols. Exogenous antioxidants like vitamin C, E, and many others cannot be synthesized by the human body and must be taken up by the diet.

## Figures and Tables

**Table 1:** Exogenous antioxidants with photoprotective or damage protective effects.

Antioxidants	Outcome of the study	Study
Ascorbic acid	Topical vitamin C 5% cream applied for six months led to clinical improvement in the appearance of photoaged skin	
	Topical vitamin C stimulates the collagen-producing activity of the dermis	
	Magnesium ascorbyl phosphate administration immediately after exposure in hairless mice significantly delayed skin tumor formation and hyperplasia induced by chronic exposure to UV radiation	Elmore, 2005 [51]
	Ascorbic acid was a photoprotectant when applied to mice and pig skin before exposure to ultraviolet (UV) radiation	Elmore, 2005 [51]

Vitamin E	UV-induced vitamin E depletion	Packer and Valacchi, 2002 [52]
	The interaction of vitamin E with the eicosanoid system may result in an anti-inflammatory effect and thereby complement the photoprotective effects of other antioxidants in the skin	Boelsma et al., 2001 [53]
	Vitamin E has skin barrier-stabilizing properties	Packer et al., 2001 [54]

Lycopene	UV light decreased skin lycopene concentrations more so than skin *β*-carotene concentrations	Ribaya-Mercadoet al., 1995 [55]
	Lycopene protects against UV-induced erythema in humans	

Carotenoids (carotene, *β*-carotene, and carotenoid mix)	Carotenoids are efficient in photoprotection, scavenging singlet oxygen, and peroxyl radicals. Supplements or a carotenoid-rich diet decreased sensitivity against UV-induced erythema	Sies and Stahl, 2004 [56]
	Supplementation with carotenoids contributes to basal protection of the skin but is not sufficient to obtain complete protection against severe UV irradiation	Stahl and Krutmann, 2006 [57]
	Dietary beta-carotene has effect on wrinkles and elasticity, procollagen gene expression, and ultraviolet (UV)-induced DNA damage in human skin	Cho et al., 2010 [58]
	Erythema-protective effect of a carotenoid mix inhibited serum lipid peroxidation	Heinrich et al., 1998 [59] Heinrich et al., 2003 [60] Lee et al., 2000 [61]
	Presupplementation with *β*-carotene before and during sunlight exposure provides protection against sunburn	Gollnick et al., 1996 [62]
	Inhibition of UV-induced epidermal damage and tumor formation in mouse models	Mathews-Roth and Krinsky, 1987 [63]

Tretinoin	Topical tretinoin ameliorates the clinical signs of photoaging	Cordero, 1983 [64] Kligman et al., 1986 [65]
	The treatment of photodamaged skin with tretinoin increased collagen I formation.	Griffiths et al., 1993 [66]
	Topical tretinoin is safe and effective in the treatment of photodamage	Gilchrest, 1997 [67]
	Improvement in photodamaged skin	Weinstein et al., 1991 [68]
	Topical tretinoin reduced the effects of photoaging	Voorhees, 1990 [69]
	Topical tretinoin in combination with sun protection as a useful approach to the treatment of sun-damaged skin	Leyden, 1998 [70]

Coenzyme Q10 (CoQ10)	Topical application of CoQ10 has the beneficial effect of preventing photoaging	Hoppe et al., 1999 [71]
	Coenzyme Q10 protects against oxidative stress-induced cell death and enhances the synthesis of basement membrane components in dermal and epidermal cells	Muta-Takada et al., 2009 [72]
	CoQ10 was shown to reduce UVA-induced MMPs in cultured human dermal fibroblasts	Inui et al., 2008 [73]

Glutathione	Glutathione is a photoprotective agent in skin cells	Connor and Wheeler, 1987 [74]

Zinc	Zn-treated fibroblasts were more resistant to UVR than cells grown in normal medium	Richard et al., 1993 [75]
	Zn can positively influence the effects of oxidative stress on cultured human retinal pigment epithelial (RPE) cells	Tate et al., 1999 [76]

Resveratrol	Application of resveratrol to the skin of hairless mice effectively prevented the UVB-induced increase in skin thickness and the development of the skin edema	Afaq and Mukhtar, 2002 [77]

Green tea	Green tea polyphenols were shown to reduce UV light-induced oxidative stress and immunosuppression	Katiyar et al., 2000 [78]
	Topical treatment or oral consumption of green tea polyphenols (GTP) inhibits chemical carcinogen- or UV radiation-induced skin carcinogenesis in different laboratory animal models	Katiyar, 2003 [79]

Green tea or caffeine	Oral administration of green tea or caffeine in amounts equivalent to three or five cups of coffee per day to UVB-exposed mice increased levels of p53, slowed cell cycling, and increased apoptotic sun burn cells in the epidermis	Lu et al., 2008 [80]

Sylimarin	Silymarin strongly prevents both photocarcinogenesis and skin tumor promotion in mice	Singh and Agarwal, 2002 [81]
	Skin cancer chemopreventive effects	Ahmad et al., 1998 [82]

Genistein	Antioxidant and anticarcinogenic effects on skin	Wei et al., 1995 [83]

Cocoa	Dietary flavanols from cocoa contribute to endogenous photoprotection, improve dermal blood circulation, and affect cosmetically relevant skin surface and hydration variables	Heinrich et al., 2006 [84]
	Photoprotection against UV-induced erythema	Heinrich et al., 2006 [84]

**Table 2:** Exogenous antioxidant's mixtures with photoprotective or damage protective effects.

Antioxidant mixtures	Outcome of the study	Study
Oral vitamin E and beta-carotene supplementation	Ultraviolet radiation-induced oxidative stress in human skin	McArdle et al., 2004 [85]
Carotenoids and tocopherols	Scavenging reactive oxygen species generated during photooxidative stress	Stahl et al., 2000 [86]
Beta-carotene, lutein, and lycopene	UV irradiation induced intensity of erythema was diminished	Albanes et al., 1996 [87]
Tomato extract and a drink containing solubilized Lyc-o-Mato	Reduction in erythema formation following UV irradiation	Aust et al., 2005 [88]
Quercetin, hesperetin and naringenin	Protective agents in certain skin diseases caused, initiated, or exacerbated by sunlight irradiation	Bonina et al., 1996 [89]
*α*-Tocopherol and ascorbate	MEDs increased markedly after intake of the combination of *α*-tocopherol and ascorbate	Fuchs and Kern, 1998 [45]
Combination of vitamins C and E	Mean MEDs increased in group receiving vitamins compared with baseline	Eberlein-Konig et al., 1998 [90]
Vitamin C, vitamin E, lycopene, beta-carotene, the rosemary polyphenol, and carnosic acid	Vitamin C, vitamin E, and carnosic acid showed photoprotective potential human dermal fibroblasts exposed to ultraviolet-A (UVA)	Offord et al., 2002 [91]
Lycopene, beta-carotene, alpha-tocopherol, and selenium	Many parameters of the epidermal defense against UV-induced damage were significantly improved	C$\overset{´}{\text{e}}$sarini et al., 2003 [92]
*β*-Carotene, lycopene, tocopherol, and ascorbic acid	Significant increase of melanin concentrations in skin was found	Postaire et al., 1997 [93]
Carotenoids (beta-carotene and lycopene), vitamins C and E, selenium, and proanthocyanidins	A selective protection of the skin against irradiation was confirmed	Greul et al., 2002 [94]

**Table 3:** Exogenous antioxidants with no protective/beneficial effects.

Antioxidant	Outcome of the study (nonbeneficial results)	Study
Lycopene	Lycopene enhances UVA-induced oxidative stress in C3H cells	Yeh et al., 2005 [95]
Carotenoids	Carotenoids were not protective against DNA lesions repairable by excision repair	Wolf et al., 1988 [96]
	No significant change in the intensity of erythema; no effects of supplementation	Garmyn et al., 1995 [97]
	No significant difference between the beta-carotene and placebo groups in incidence of cancer	Green et al., 1999 [98]
	No significant effect of *β*-carotene on either number or time of occurrence of new nonmelanoma skin cancer	Greenberg et al., 1990 [99]
	An average of 12 years of supplementation with beta-carotene does not affect the development of a first NMSC	Frieling et al., 2000 [100]
	Supplementation with *β*-carotene produced no reduction of the incidence of malignant neoplasms	Hennekens et al., 1996 [101]
